# Maternal Cigarette Smoke Exposure Contributes to Glucose Intolerance and Decreased Brain Insulin Action in Mice Offspring Independent of Maternal Diet

**DOI:** 10.1371/journal.pone.0027260

**Published:** 2011-11-04

**Authors:** Hui Chen, Miguel A. Iglesias, Vanni Caruso, Margaret J. Morris

**Affiliations:** 1 School of Medical and Molecular Bioscience, Faculty of Science, University of Technology, Sydney, Sydney, New South Wales, Australia; 2 Department of Pharmacology, School of Medical Sciences, University of New South Wales, Sydney, New South Wales, Australia; 3 Discipline of Physiology, School of Medical Sciences, The University of Sydney, Sydney, New South Wales, Australia; Sapienza University of Rome, Italy

## Abstract

**Background:**

Maternal smoking leads to intrauterine undernutrition and is associated with low birthweight and higher risk of offspring obesity. Intrauterine smoke exposure (SE) may alter neuroendocrine mediators regulating energy homeostasis as chemicals in cigarette smoke can reach the fetus. Maternal high-fat diet (HFD) consumption causes fetal overnutrition; however, combined effects of HFD and SE are unknown. Thus we investigated the impact of combined maternal HFD and SE on adiposity and energy metabolism in offspring.

**Method:**

Female Balb/c mice had SE (2 cigarettes/day, 5 days/week) or were sham exposed for 5 weeks before mating. Half of each group was fed HFD (33% fat) versus chow as control. The same treatment continued throughout gestation and lactation. Female offspring were fed chow after weaning and sacrificed at 12 weeks.

**Results:**

Birthweights were similar across maternal groups. Faster growth was evident in pups from SE and/or HFD dams before weaning. At 12 weeks, offspring from HFD-fed dams were significantly heavier than those from chow-fed dams (chow-sham 17.6±0.3 g; chow-SE 17.8±0.2 g; HFD-sham 18.7±0.3 g; HFD-SE 18.8±0.4 g, *P*<0.05 maternal diet effect); fat mass was significantly greater in offspring from chow+SE, HFD+SE and HFD+sham dams. Both maternal HFD and SE affected brain lactate transport. Glucose intolerance and impaired brain response to insulin were observed in SE offspring, and this was aggravated by maternal HFD consumption.

**Conclusion:**

While maternal HFD led to increased body weight in offspring, maternal SE independently programmed adverse health outcomes in offspring. A smoke free environment and healthy diet during pregnancy is desirable to optimize offspring health.

## Introduction

Childhood obesity is currently a major health problem within the global obesity pandemic, with 22 million children under five estimated as overweight/obese [1]. In the long term, 50% of obese adolescents will remain obese in adulthood [2], increasing their risk of developing associated insulin resistance and cardiovascular disease, adding to the already enormous cost of obesity related diseases. Unhealthy maternal nutrition during gestation, including both under- and over-nutrition, is critical in predisposing the offspring to obesity and related disorders during postnatal development [3], [4].

While maternal obesity represents a common threat to the wellbeing of the next generation, intrauterine undernutrition is not uncommon. In western countries, maternal smoking during pregnancy is a major cause of intrauterine undernutrition, leading to low body weight [5], [6] and head circumference [6], [7] at birth. A number of studies have revealed a strong inverse relationship between birthweight and the risk of developing abdominal obesity, hypertension, and metabolic disorders from childhood [8], [9], [10], [11]. About 20–50% of women report smoking at the onset of pregnancy [12], with 25–29% of women arriving at the end of their pregnancy without stopping smoking, and 50% of non-smoking mothers were exposed to passive smoking during pregnancy [13].

There are several theories underlying the problem of lower birth weight. Firstly, vascular changes due to nicotine and carbon monoxide in the mothers could limit nutrient delivery to the fetus to restrict intrauterine growth. Secondly, catecholamines released from the adrenal glands and nerve cells due to smoke exposure (SE) can cause vasoconstriction to limit placental blood flow, restricting intrauterine nutrition [14]. In response to undernutrition, nutrients are selectively distributed to preserve brain growth, by reducing supply to other less vital organs. These adaptations limit cell numbers in key organs, such as liver and muscle, altering glucose and fat metabolism [15], increasing the prevalence of type 2 diabetes [15]. Indeed, maternal smoking is associated with increased risk of both childhood and adulthood obesity in offspring [16], [17], [18], [19], [20], [21].

However, the impact of maternal smoking on offspring is likely more than just intrauterine undernutrition caused by placental limitation. We previously showed that both short- and long-term smoke exposure (SE) leads to reduced energy intake and weight gain in normal mice [22], [23], [24]. Many energy homeostatic regulators, such as the appetite-stimulant neuropeptide Y (NPY), the anorexigenic hormone leptin, and uncoupling proteins, were found to be changed by SE [23]. The addictive substance of cigarette smoke, nicotine, can accumulate in the fetus, which was shown to change the chemicals involved in its energy homeostasis [25], leading to low birth weight. Products of cigarette smoke, such as carbon monoxide, can also directly affect the fetal brain and organs [14], which may have a major impact on fetal development, in addition to that of nicotine. Therefore, the means by which maternal smoking affects the energy metabolism in offspring remains unclear.

Both active and passive smoking have been reported to contribute to glucose intolerance and insulin resistance, leading to type 2 diabetes [26], [27]. Increased glucose intolerance was also reported in offspring of smoking mothers. However, it is unknown whether this is a direct outcome of maternal smoking during pregnancy or secondary to their own unhealthy eating habits and lifestyle, as offspring from smoking parents are more likely to consume a poor diet and be inactive [5], [28], [29], [30]. Nevertheless, maternal smoking can lead to obesity and related disorders, such as insulin resistance and glucose intolerance, in both childhood and adulthood [4], [8], [9], [10], [11], [31], [32]. Insulin receptors are widely distributed in the brain and concentrated in the appetite regulating centre, the hypothalamus [33], exerting both central and peripheral effects. Brain insulin infusion can reduce food intake and body weight in a dose-dependent manner, while injection of insulin antibodies exerts opposite effects [34], [35]. This is because insulin and the anorexic hormone leptin share common intracellular signaling pathways via insulin receptor substrate and the enzyme phosphoinositide 3-kinase [36], enabling cross talk effects on feeding regulation. More interestingly, brain insulin signaling is required to inhibit glucose synthesis in the liver [37], and glucose metabolism within the brain can also regulate blood lipid levels and subsequent insulin sensitivity [38]. This suggests that any dysfunction of brain insulin action due to altered signaling pathway or brain glucose metabolic orders can contribute to hyperglycemia and subsequent glucose intolerance, one of the first manifestations towards the development of Type 2 diabetes. However, none of the previous studies has drawn a connection between maternal smoking and programming of abnormal brain insulin response that leads to obesity and glucose intolerance phenotype in offspring.

On the other hand, maternal high-fat diet (HFD) consumption is known to cause intrauterine overnutrition, which has been shown to program glucose intolerance in offspring even when they consumed low fat diet [32]. Surprisingly, infant growth retardation was reported in some obese mothers smoking during pregnancy [39]. This suggests that HFD consumption cannot always reverse the intrauterine undernutrition due to maternal smoking. However, to date no work has systematically studied the effects of maternal cigarette SE in combination with HFD consumption on the adiposity and metabolic changes in offspring. We postulated that when obesity and smoking coincide during pregnancy, they may have additive deleterious effects on fetal energy homeostasis.

It has been reported that maternal smoking has no impact on the body weight of female offspring [18]. However, the metabolic changes in the female offspring remain largely unknown, thus in this study female offspring were examined. We hypothesized that long-term maternal SE prior to and during gestation would alter hypothalamic appetite regulators, especially their response to insulin challenge, to promote hyperphagia and adiposity in female offspring. We also hypothesized that maternal HFD feeding would exaggerate the detrimental impact of maternal SE on offspring. By feeding the offspring with balanced low fat diet, the intrauterine impact on adiposity and glucose metabolism can be distinguished from that from unhealthy dietary habit.

## Materials and Methods

### 1. Ethics Statement

This study was approved by the Animal Care and Ethics Committee of the University of New South Wales (ACEC #06/61B).

### 2. Animals

Female Balb/c mice (n = 36, 7 weeks, Animal Resources Centre, Perth, Australia) were housed at 20±2°C, and maintained on a 12∶12 h light/dark cycle (lights on 06:00 h). One group of mice were fed standard laboratory chow (11 kJ/g, 14% fat, 21% protein, 65% carbohydrate, Gordon's Specialty Stockfeeds, NSW, Australia), while the second group was fed a palatable cafeteria style HFD providing 15.33 kJ/g (34% fat, 19% protein, 47% carbohydrate) as described previously [32], [40]. Within each dietary cohort, half the mice were exposed to cigarette smoke (SE, 2 cigarettes/day, 5 days/week, nicotine≤1.2 mg, CO≤15 mg, Philip Morris, VIC, Australia) inside a perspex chamber for 30 min. The control sham exposed mice were put in an identical chamber for the same period. This yielded 4 maternal groups, chow+sham, chow+SE, HFD+sham, and HFD+SE. The offspring from these 4 maternal groups were named chow-sham, chow-SE, HFD-sham, and HFD-SE, respectively.

Dietary and SE interventions continued for 5 weeks before females were mated with male mice (9 weeks) from the same source. The same treatment continued throughout gestation and lactation. During lactation the offspring remained in the home cage without SE. Pups were weighed every 4 days and weaned at postnatal day 20. Female breeders were killed by anesthetic overdose (ketamine/xylazine 180/32 mg/kg, i.p.) 1 day after pups were weaned. All female offspring were fed chow after weaning and sacrificed at 12 weeks. Energy intake and body weight were recorded weekly after weaning.

### 3. Offspring IP glucose tolerance test (IPGTT)

At 11 weeks of age, an IPGTT was performed. Animals were fasted for 5 h and weighed. A blood sample was collected from the tail tip to establish baseline glucose level at T_0_ by glucose meter (Accu-Chek®, Roche Diagnostics, Nutley, USA). Mice were then administered 2 g glucose/kg body weight (i.p.). Blood glucose levels were measured at 15, 30, 60, and 90 min and expressed as glucose level above baseline (T_0_). Area under the curve (AUC) was calculated for each mouse.

### 4. Endpoint insulin injection and sample collection

At 12 weeks, 5 h-fasted female offspring were deeply anesthetized (ketamine/xylazine 75/10 mg/kg, i.p.). Within each group, half of the mice received a single dose of insulin (1 U/kg i.p, Actrapid, short-acting human insulin, Novo-Nordisc Pharmaceuticals Pty. Ltd, Bagsvrd, Denmark) while the other half received saline control. Offspring were culled 10 min post-injection.

After measurement of naso-anal (N-A) length, blood was collected, plasma separated immediately and stored at −20°C for leptin and insulin measurements. Then animals were killed by decapitation. The whole hypothalamus was dissected, snap frozen in liquid nitrogen, and stored at −80°C for measuring mRNA expression of genes of interest. Body fat (gonadal, retroperitoneal (Rp), and mesenteric) was dissected and weighed, as well as organs (heart, liver, and kidney) and skeletal muscle (soleus, extensor digitorum longus (EDL), and tibialis). Rp fat and liver were kept to provide markers of metabolism. Tibia length was measured as a marker of growth.

### 5. Leptin and insulin assays

Plasma leptin and insulin were measured using radioimmunoassay kits (Linco, St. Charles, Missouri, USA) with detection limits of 0.5 and 0.05 ng/ml respectively. Intra- and inter- leptin assay coefficients of variation of were 3.3% and 4.1% respectively. Intra- and inter- insulin assay coefficient of variation were 2.2% and 8.9%, respectively.

### 6. Western blotting

Proteins were isolated from liver as previously described [31]. Protein concentration was determined by Bradford protein assay (Bio-Rad Inc, Hercules, CA, USA) using BSA as standard. Proteins (50 µg) were then separated on a polyacrylamide gel and transferred to polyvinylidine fluoride membrane. Membranes were incubated overnight at 4°C with the primary antibody for target and housekeeping proteins (phosphorylated-protein kinase B (PKB) Ser473, phosphorylated-glycogen synthase kinase-3β (GSK-3β), Cell Signaling, Beverly, MA, USA; housekeeping β-actin, Sigma, St. Louis, MO, USA), followed by secondary antibody (Anti-rabbit IgG, HRP-linked Antibody, Cell Signaling, Beverly, MA, USA) for 2 h. Protein expression was detected on medical X-Ray film (SuperRX, Fujifilm Corporation, Tokyo, Japan), developed and fixed using an automatic lightweight film processor (CP1000, AGFA HealthCare NV B-2640 Mortsel, Belgium). Protein band density was determined by scanning (ChemiDoc XRS Bio-Rad Inc, Hercules, CA, USA) and quantified using Bio-Rad Laboratories Quantity One 1-D Analysis Software. Results are expressed in arbitrary units.

### 7. Quantitative real-time PCR

Total RNA was isolated from individual hypothalamus using TriZol reagent (Invitrogen Australia Pty Limited, Melbourne, VIC, Australia) according to manufacturer's instructions. Purified total RNA was used as a template to generate first-strand cDNA using M-MLV Reverse Transcriptase, RNase H-, Point Mutant Kit (Promega, Madison, WI, USA). Pre-optimized probe/primers (Applied Biosystem, Foster City, CA, USA) were used for real-time PCR (Realplex2, Eppendorf AG, Hamburg, Germany). Probe sequences from 5′→3′ offered by manufactory and NCBI Gene References are, adipose triglyceride lipase (ATGL): GCCTGCCTGGGTGAAGCAGGTGCCA (NM_025802.2), Carnitine palmitoyl-transferase (CPT)-1α: ACCGTGAGCAGGTACCTGGAGTCTG (NM_013495.2), leptin: ACACACGCAGTCGGTATCCGCCAAG (NM_008493.3), monocarboxylate transporter (MCT) 2: CCAGCTCCTTCACCAGCTCCCTAAG (NM_009197.2), MCT4: CCACCAATAGCAGGCTGGATATATG (NM_146136.1), NPY: ATCTCATCACCAGACAGAGATATGG (NM_023456.2), proopiomelanocortin (POMC): AACCTGCTGGCTTGCATCCGGGCTT (NM_008895.3), single-minded gene (Sim)1: TCTTTCCAGAAGGGCTTGGCGAGGC (NM_011376.3), TNFα: CCCTCACACTCAGATCATCTTCTCA (NM_013693.2), Y1 receptor: ATATTCATATGCTACTTCAAGATAT (NM_010934.3).

The target gene probes were labeled with FAM and housekeeping gene 18s rRNA was labeled with VIC. Genes of interest were quantified in a single multiplexing reaction, and standardized to housekeeping genes. An individual sample from the control group was assigned as calibrator against which all other samples were expressed as fold difference. The results were further confirmed by housekeeping gene GAPDH labeled by VIC.

### 8. Statistical methods

Results are expressed as mean±SEM. Data were tested for normality (Graphpad Prism 5, La Jolla, CA, USA). Parameters not normally distributed (N-A length, mesenteric fat mass, plasma leptin and insulin concentrations, UCP1, and leptin mRNA expression) were log transformed to normal distribution and then analyzed. Body weight of pups over time was analyzed using ANOVA with repeated measures, followed by post hoc Fisher's Least Significance Difference (LSD) test. Differences in fat and organ weights, blood and plasma hormone concentrations, mRNA and protein expression in all tissues were analyzed using two-way ANOVA followed by a post hoc LSD test. *P*<0.05 was considered significant.

## Results

### 1. Effects of interventions on the breeders

The four groups of female breeders started at similar body weights (Table 1). Mice fed a HFD consumed 33% and 45% more calories in sham and SE groups, respectively (*P*<0.05, HFD effect). After 5 weeks of intervention, those fed HFD were significantly heavier than chow-fed females (*P*<0.05, HFD+sham *vs.* chow+sham, HFD+SE *vs.* chow+SE), while SE mice were significantly lighter than sham-exposed mice (*P*<0.05, chow+SE *vs.* chow+sham, HFD+SE *vs.* HFD+sham; Table 1). At the conclusion of the experiment, HFD-fed mice also had greater adiposity in all locations sampled, whereas SE only significantly reduced the ovarian fat mass in chow-fed mice (*P*<0.05, chow+SE *vs.* chow+sham; Table 1). HFD consumption only significantly increased liver weight, while SE markedly reduced both liver and kidney weights (*P*<0.05, SE effect). Blood glucose level was only reduced in chow+SE compared with chow+sham dams (*P*<0.05, Table 1).

**Table 1:** Parameter of the female breeders.

Breeders	chow+sham	chow+SE	HFD+sham	HFD+SE
	n = 11	n = 11	n = 11	n = 11
**BW pre-treatment (g)**	18.8±0.2	18.7±0.4	17.6±0.3	17.9±0.3
**BW pre-pregnancy (g)**	20.5±0.2	19.0±0.2#	23.3±0.4*	21.2±0.4# *
**Energy intake (kJ/24 h)**	38.0±1.5	36.3±1.5	50.8±4.1*	52.8±4.9*
**Rp fat (mg)**	30.2±14.2	16.6±5.4	196.6±27.8*	200.7±52.1*
**Gonadal fat (mg)**	240.0±87.3	122.6±28.0#	1026.2±158.4*	993.5±248.4*
**Mesenteric fat (mg)**	452.8±25.4	368.9±33.5	599.4±36.9*	618.9±68.6*
**Liver (mg)**	1484.5±55.0	1066.1±53.7#	1640.5±174.8*	1292.5±46.5# *
**Kidney (mg)**	154.5±3.8	138.1±4.32#	145.9±4.6	141.0±3.7
**Blood glucose (mmol/l)**	9.25±0.52	6.11±0.41#	9.53±0.80	8.13±1.00

# , P<0.05, SE effect;

*, P<0.05, HFD effect. Results are expressed as mean ± SE. Data were analysed by two-way ANOVA, followed by post hoc LSD tests.

BW: body weight; Rp: retroperitoneal.

### 2. Effects of maternal intervention on offspring

#### 2.1 Growth and adiposity

**Figure 1:**
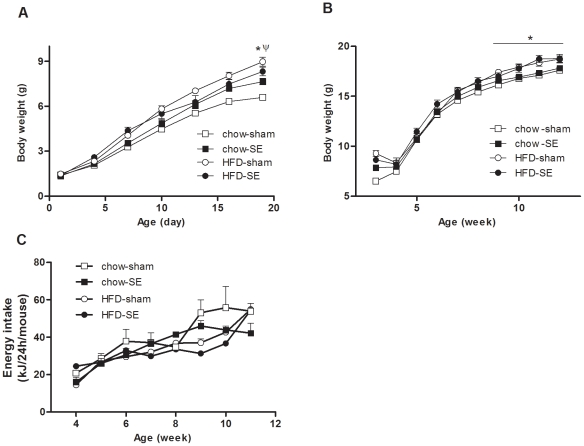
Body weight changes of the offspring mice. Body weight changes during the suckling and post-weaning periods in chow-sham (open square n = 24), chow-SE (solid square n = 21), HFD-sham (open circle n = 17), and HFD-SE (solid circle n = 15) groups. Results are expressed as mean ± SEM. Data were analysed by ANOVA with repeated measures followed by a post hoc LSD test. *, *P*<0.05, maternal HFD effect. Ψ, *P*<0.05, chow-sham significantly different from the other 3 groups. Chow-sham: offspring from dam fed chow & sham exposed; chow-SE: offspring from dam fed chow & cigarette smoke exposed; HFD-sham: offspring from dam fed HFD & sham exposed; HFD-SE: offspring from dams fed HFD & cigarette smoke exposed.

Although pups born of SE dams (chow-SE and HFD-SE) appeared smaller than those from sham-exposed dams, this did not reach statistical significance (Table 2). However, after birth mice from dams exposed to either HFD or cigarette smoke grew faster than those from the control dams (chow-sham, Figure 1A). As a result, at weaning, pups in chow-SE, HFD-sham and HFD-SE groups were 15%, 36% and 26% heavier than the chow-sham group, respectively, despite all groups consuming standard rodent chow. From 9 weeks, offspring from HFD-fed mothers (HFD-sham & HFD-SE groups) became significantly heavier than those from chow-fed mothers (*P*<0.05, maternal HFD effect, Figure 1B). At 12 weeks, average body weight of chow-sham mice was 7–10% smaller than the other 3 groups, and fat mass (Rp, gonadal, and mesenteric pads) was also significantly smaller in chow-sham mice compared with the other 3 groups (*P*<0.05, Table 2). After standardizing by body weight, only gonadal fat remained significantly different. Organ weights, including liver, kidney and heart, showed a similar pattern as fat mass, with chow-sham offspring having the smallest organ masses (*P*<0.05, chow-sham *vs.* the other 3 groups, Table 2). Skeletal muscle mass (soleus and EDL) were significantly greater in mice from HFD-fed dams (*P*<0.05, maternal HFD effect, Table 2). However, all these differences in organ and muscle mass disappeared when data were standardized by body weight, suggesting that the increase was proportional to body weight. The body length reflected by N-A and tibia length was not significantly different between groups. Interestingly, the daily energy intake in the chow-sham group started to increase markedly from 9 weeks (Figure 1C), and over the whole post-weaning period it was greater than the other 3 groups, which were at a similar level (*P*<0.05, chow-sham *vs.* all other groups, Table 2).

**Table 2:** Parameters of the female offspring.

Offspring	chow-sham	chow-SE	HFD-sham	HFD-SE
	n = 24	n = 21	n = 17	n = 15
**BW at 1 day (g)**	1.45±0.12	1.35±0.06	1.48±0.15	1.38±0.07
**BW at 20 days (g)**	6.49±0.14Ψ	7.83±0.2	9.27±0.3	8.62±0.29
**BW at 12 weeks (g)**	16.3±0.2Ψ	17.4±0.2	17.9±0.3	17.4±0.5
**Energy intake (kJ/24 h)**	40.2±4.5Ψ	35.3±3.7	34.3±4.2	33.7±3.3
**N-A length (cm)**	8.57±0.15	8.92±0.05	8.76±0.07	8.84±0.06
**Tibia length (cm)**	1.93±0.02	1.90±0.02	1.97±0.01	1.96±0.01
**Mesenteric fat (mg)**	336.6±7.6Ψ	376.1±10.3	404.8±36.9	378.7±16.8
**Gonadal fat (mg)**	295.0±7.66Ψ	384.9±22.0	404.8±36.9	373.7±27.7
**Rp fat (mg)**	72.2±3.4Ψ	85.9±3.6	103.5±5.8	96.1±6.9
**Liver (mg)**	646.6±13.2Ψ	673.6±12.2	718.9±17.7	693.9±18.8
**Kidney (mg)**	92.0±1.7Ψ	98.6±1.9	99.8±2.1	98.0±2.7
**Heart (mg)**	84.5±2.0Ψ	91.1±1.7	94.0±1.7	90.9±1.4
**EDL (mg)**	13.1±0.30	13.3±0.4	14.2±0.4*	14.4±0.6*
**Soleus (mg)**	9.15±0.19	10.1±0.21#	11.1±0.50*	9.93±0.32#
**Tibialis (mg)**	64.4±0.8	67.8±3.0	69.1±1.8	66.8±1.9
**Plasma insulin (ng/ml)** †	0.07±0.02	0.09±0.02	0.04±0.01	0.07±0.03

Results are expressed as mean ± SEM († n = 6–10). Data were analysed by two-way ANOVA, followed by post hoc LSD tests.

Ψ *P*<0.05, different from the other 3 groups;

*P<0.05, maternal HFD effect;

# *P*<0.05, maternal SE effect.

BW: body weight; EDL: extensor digitorum longus; N-A: naso-anus; Rp: retroperitoneal.

**Figure 2:**
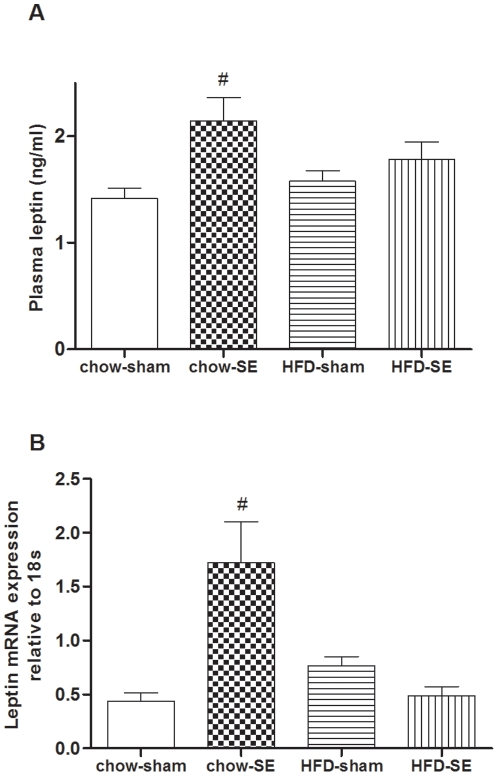
Plasma level and fat mRNA expression of leptin. Plasma leptin (n = 15–24) and leptin mRNA expression in Rp fat (n = 10–12). Results are expressed as mean ± SEM. Data were analysed by two-way ANOVA followed by a post hoc LSD test. #, *P*<0.05, significantly different from chow-sham group. Chow-sham: offspring from dam fed chow & sham exposed; chow-SE: offspring from dam fed chow & cigarette smoke exposed; HFD-sham: offspring from dam fed HFD & sham exposed; HFD-SE: offspring from dams fed HFD & cigarette smoke exposed.

Concentrations of the adipose hormone leptin were only significantly altered in chow-SE mice, which were 50% higher than chow-sham mice (*P*<0.05, Figure 2A). This coincided with significantly upregulated Rp leptin mRNA expression (*P*<0.05, Figure 2B).

#### 2.2 Glucose homeostasis

**Figure 3:**
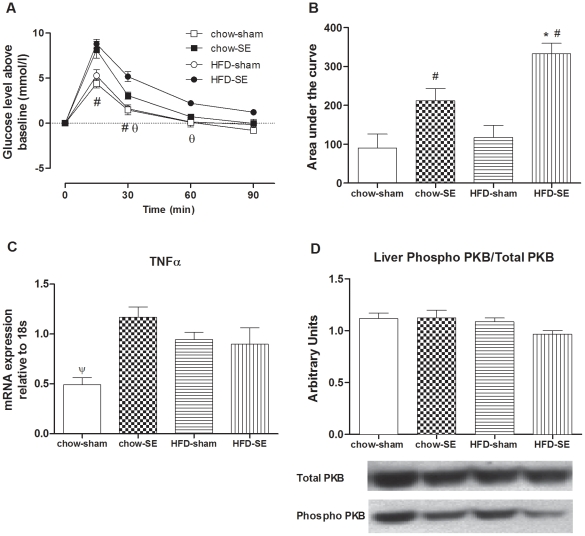
Glucose tolerance and markers related to insulin sensitivity. (A) Change in blood glucose levels above the baseline values during an IPGTT at 11 weeks (glucose 2 g/kg, n = 6) in chow-sham (open square), chow-SE (solid square), HFD-sham (open circle), and HFD-SE (solid circle) pups. Results are expressed as mean ± S.E.M. Data were analysed by ANOVA with repeated measures followed by a post hoc LSD test. Area under the curve for (A) is shown in (B). (C) TNFα mRNA expression in the Rp fat at 12 weeks (n = 12). (D) Liver phosphorylated-PKB/total PKB protein 10 min after exogenous insulin injection (1 U/kg, ip) at 12 weeks (n = 5–6) and representative blots. Results are expressed as mean ± SEM. Data in (B), (C), and (D) were analysed by two-way ANOVA followed by post hoc LSD tests. (A) #, *P*<0.05, maternal SE effect, HFD-SE & chow-SE different from HFD-sham & chow-sham, respectively. θ, *P*<0.05, HFD-SE different from chow-SE. (B, C) #, *P*<0.05, maternal SE effect; ψ, *P*<0.05, chow-sham different from the other 3 groups. Chow-sham: offspring from dam fed chow & sham exposed; chow-SE: offspring from dam fed chow & cigarette smoke exposed; HFD-sham: offspring from dam fed HFD & sham exposed; HFD-SE: offspring from dams fed HFD & cigarette smoke exposed.

While basal blood glucose (T_0_ of IPGTT) was lower in offspring of HFD+SE dams (4.35±0.25 mM vs. 6.17±0.16 mM in HFD-sham group, *P*<0.05, maternal SE effect), it was similar between chow-sham (6.67±0.31 mM) and chow-SE (5.93±0.13 mM) groups. When standardized by basal levels, at 15 and 30 min post-glucose injection, mice from SE dams had significantly greater increments in glucose (chow-SE +8.13±0.94 mM, HFD-SE +8.80±0.51 mM) than those from sham-exposed dams (chow-sham +4.40±0.48 mM, HFD-sham +5.28±0.66 mM, *P*<0.05, maternal SE effect, Figure 3A); glucose levels in HFD-SE mice remained significantly higher than chow-SE group until 60 min post-injection (*P*<0.05, maternal HFD effect). These differences were also reflected in the AUC (*P*<0.05, maternal SE & HFD effects, Figure 3B). At 90 min post-injection, the blood glucose levels in chow-sham, chow-SE, and HFD-sham groups had returned to baseline, but not in the HFD-SE group.

At 12 weeks, plasma insulin concentration was not significantly different between groups (*P*<0.05, Table 2). Rp TNFα mRNA expression was significantly increased in the chow-SE, HFD-sham and HFD-SE groups compared with chow-sham group (*P*<0.05, Figure 3C). In liver, upon exogenous insulin stimulation, phosphorylated-PKB levels tended to be decreased in the HFD-SE group, as reflected in a decreased ratio of phosphorylated/total PKB although not reaching statistical significance (Figure 3D).

#### 2.3 Energy homeostasis regulators in the hypothalamus

**Figure 4:**
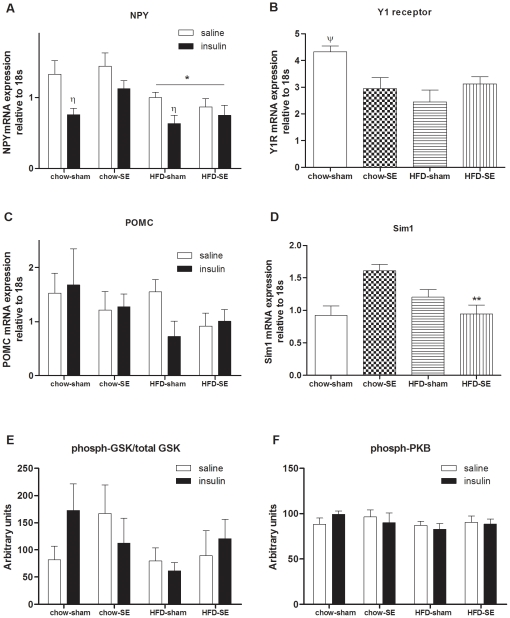
mRNA and protein expression in the hypothalamus. mRNA expression of NPY (A), Y1 receptor (B), POMC (C), and Sim1 (D), and phosphorylated/total GSK protein (E) and phosphorylated-PKB protein (F) before and 10 min after exogenous insulin injection (1 U/kg, ip) in the offspring hypothalamus at 12 weeks (n = 8–9). Results are expressed as mean ± SEM. Data in (A) and (C) were analysed by multiple-factor ANOVA followed by post hoc LSD tests. Data in (B) and (D) were analysed by two-way ANOVA followed by post hoc LSD tests. *, *P*<0.05, maternal HFD effect; **, *P*<0.05, significantly different from chow-SE group; η, P<0.05, insulin injection effect. (A) saline injection (open bars), insulin injection (1 U/kg, closed bars). Chow-sham: offspring from dam fed chow & sham exposed; chow-SE: offspring from dam fed chow & cigarette smoke exposed; HFD-sham: offspring from dam fed HFD & sham exposed; HFD-SE: offspring from dams fed HFD & cigarette smoke exposed. NPY: neuropeptide Y; POMC: proopiomelanocortin; Sim1: single-minded gene 1.

Maternal obesity regulated hypothalamic NPY mRNA, which was lower in offspring from HFD+sham and HFD+SE dams (*P*<0.05 maternal HFD effect, Figure 4A), and Y1 receptor mRNA was reduced in offspring from the maternal groups with interventions (chow-SE, HFD-sham, HFD-SE pups, Figure 4B), in line with their lower average energy intake over the whole postweaning period shown in Table 2. Hypothalamic POMC mRNA expression was not different among the 4 groups (Figure 4C), while amongst the SE offspring, Sim1 mRNA was significantly reduced by HFD (chow-SE *vs*. HFD-SE groups; Figure 4D).

Phosphorylated (P) PKB and P/total GSK-3β, were similar between groups (Figure 4E,F). Ten minutes after a peripheral injection of insulin, these proteins were not significantly increased (Figure 4E,F). However, hypothalamic NPY mRNA expression in the chow-sham and HFD-sham groups was significantly reduced (*P*<0.05, insulin effect, Figure 4A), but this was not observed in the offspring from SE dams. POMC mRNA expression was not significantly changed after insulin injection (Figure 4C).

**Figure 5:**
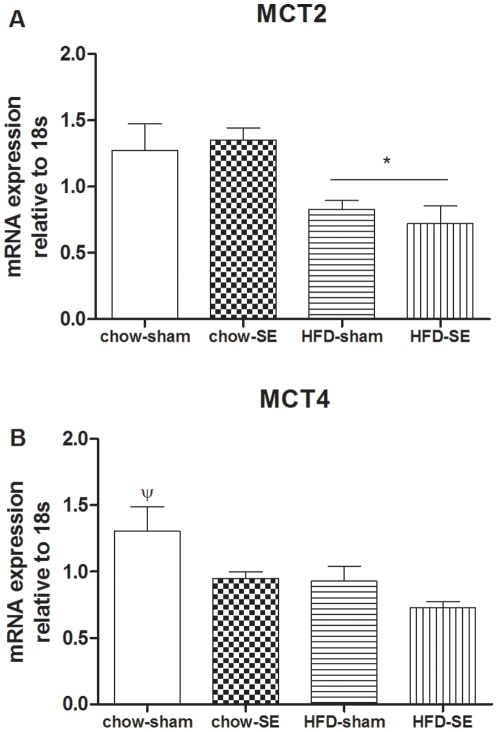
mRNA expression of MCTs in the hypothalamus. mRNA expression of MCT2 (A), and MCT4 (B) in the offspring hypothalamus at 12 weeks (n = 8–9). Results are expressed as mean ± SEM. Data were analysed by two-way ANOVA followed by a post hoc LSD tests. *, *P*<0.05, maternal HFD effect; ψ, *P*<0.05, significantly different from all the other 3 groups. Chow-sham: offspring from dam fed chow & sham exposed; chow-SE: offspring from dam fed chow & cigarette smoke exposed; HFD-sham: offspring from dam fed HFD & sham exposed; HFD-SE: offspring from dams fed HFD & cigarette smoke exposed.

Hypothalamic MCT2 mRNA was downregulated in offspring from HFD-fed dams (*P*<0.05, maternal HFD effect, Figure 5A), while MCT4 was reduced in both HFD-fed and SE groups (*P*<0.05, maternal HFD and SE effects, Figure 5B).

#### 2.4 Metabolic markers in the Rp fat

**Figure 6:**
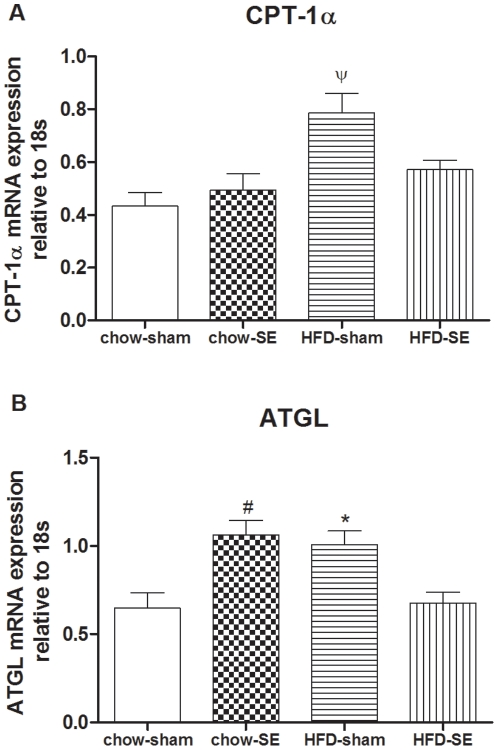
mRNA expression of CPT-1 α and ATGL in the fat. mRNA expression of CPT-1α (A), and ATGL (B) in the Rp fat at 12 weeks (n = 8–9). Results are expressed as mean ± SEM. Data were analysed by two-way ANOVA followed by post hoc LSD tests. *, *P*<0.05, maternal HFD effect; #, P<0.05, maternal SE effect; ψ, *P*<0.05, significantly different from the other 3 groups. Chow-sham: offspring from dam fed chow & sham exposed; chow-SE: offspring from dam fed chow & cigarette smoke exposed; HFD-sham: offspring from dam fed HFD & sham exposed; HFD-SE: offspring from dams fed HFD & cigarette smoke exposed.

CPT-1α mRNA expression was significantly increased in the HFD-sham group compared with all other groups when standardized to 18s (P<0.05, Figure 6A), Rp ATGL mRNA expression was upregulated in the chow-SE and HFD-sham groups (P<0.05 compared with chow-sham mice, Figure 6B), but such adaptation disappeared when maternal HFD consumption and SE were combined. Similar relative changes were observed in mRNA expression when GAPDH was used as housekeeping gene, although the magnitude of the increase in CPT-1α was lower (data not shown).

## Discussion

The current study investigated two factors that may coexist during gestation in western society, cigarette SE and HFD-consumption. It revealed that maternal SE alone only led to faster weight gain during the suckling period while maternal HFD-consumption led to greater body weight gain throughout the lifespan of the offspring, from the suckling period to adulthood. However, maternal SE appeared to be an independent factor to determine glucose intolerance and some abnormal brain responses to insulin in offspring. The addition of maternal HFD consumption exaggerated the degree of glucose intolerance in offspring, enhancing the risk of them developing metabolic disorders.

Maternal nutrition during gestation can directly impact the wellbeing of the offspring in both childhood and adulthood. Smoking is an addictive behaviour with low cessation rate, and repeated relapse on cessation is common [41]. Difficulty controlling weight gain upon smoking cessation is another reason preventing people from quitting [42], [43]. As a result, a significant number of women do not stop smoking even during pregnancy. This markedly compromises the health of the offspring as shown in the current study and by others [17], [20], [21], [44], [45], [46], [47], [48], [49].

Low birthweight is commonly associated with maternal smoking [7], [25], [50], and we observed this trend in pups from SE dams, without statistical significance. This may be related to the relatively low cigarette dose employed in the current study, which may also contribute to the unaltered daily energy intake of the dams, especially in HFD-fed mice [40]. We did observe smaller body weight in both chow+SE and HFD+SE breeders, suggesting that SE can affect body weight without any impact on food intake, as observed in humans [51]. The lower maternal body weight could be due to increased energy expenditure observed in our previous studies [22], [23], [24], which may have affected fetal growth. It also has to be considered that smoking *per se* is a predisposing factor for abdominal obesity when dietary fat is not restricted [52], as shown by our previous study [53] and in the dams here. In HFD+SE dams, the Rp and mesenteric fat masses were unchanged compared with HFD+sham dams, suggesting that loss of lean body mass contributed to the smaller body weights. Smoking is strongly associated with wasting [54] due to increased protein turnover [55]. While muscle loss is one of the major contributors [54], reduced mass of other organs may also play a role, such as the lower kidney and liver weights in SE dams observed in this study. SE in the HFD-fed dams reduced the liver weight gain, associated with lipid accumulation due to HFD [56]. With increased adiposity in the dams, the fetuses would suffer from the detrimental impact from both maternal SE and obesity; both of these factors alone are known to cause various metabolic disorders in offspring.

Following intrauterine growth retardation linked to SE, faster postnatal growth is commonly observed in children, leading to childhood obesity [5], [28], [57]. In the current study, significantly faster growth was displayed in offspring with maternal SE and/or obesity, suggesting that any negative intrauterine environment has the capacity to disturb normal postnatal development. As a result, all of these offspring had significantly greater adiposity. Adipose CPT-1α and ATGL mRNA expression were increased in chow-SE and/or HFD-sham groups respectively, suggesting enhanced fatty acid oxidation and lipolysis. This could be an adaptation in response to the faster growth, but not sufficient to reverse the increased adiposity. However, such adaptation disappeared in the HFD-SE group, demonstrating an enhanced metabolic risk when maternal obesity and SE were combined.

Both active and passive smoking contribute to glucose intolerance and insulin resistance, leading to type 2 diabetes [26], [27]. The fact that glucose intolerance was displayed in mice from SE dams regardless of maternal diet, suggests that maternal smoking has a very strong influence on the glucose metabolism of offspring. With fetal nicotine concentrations generally being 15% above maternal levels [58], it is very likely that cigarette smoke inhaled by pregnant mothers can directly affect fetal organs involved in glucose disposal and insulin sensitivity, explaining the glucose intolerance observed in the offspring of SE dams. Recent studies also showed an increased pancreatic beta-cell apoptosis and changes in gene expression following fetal and neonatal exposure to nicotine [46], [49]. It has been speculated that the impaired insulin sensitivity among smokers may be due to nicotine, carbon monoxide, or other agents in tobacco smoke [59]. In addition, visceral obesity caused by smoking is also thought to be a key contributor even in children [60]. TNFα is one of the inflammatory cytokines directly causing hepatic insulin resistance. All these contributing factors found in humans were present in the offspring from SE dams in the current study, possibly leading to their impaired insulin action. Lower baseline insulin concentration at 12 weeks in the HFD-sham group is different from our previous findings in the rat [32]. It has been shown that maternal HFD consumption impairs β-islet function in offspring [61], which may lead to low baseline insulin concentration at some stage of development. Low insulin release may directly lead to delayed glucose clearance during IPGTT in offspring from HFD+SE dams compared with those from chow+SE dams.

In the current study, impairment in the response to insulin was evident in the hypothalamus. Interestingly, insulin receptors and signaling pathways are present in the hypothalamus, specifically in the arcuate nucleus, where NPY and POMC are predominantly synthesized [62], [63], and in mice fed HFD the defect in hypothalamic insulin action precedes liver insulin resistance [64]. Central insulin signaling has been shown to reduce NPY and increase POMC expression in the hypothalamus [65]. Although the phosphorylation of PKB and GSK3β protein were not changed in the offspring of SE dams, ten minutes after exogenous insulin injection, NPY mRNA expression was significantly downregulated in chow-sham and HFD-sham groups. The amount of insulin that crossed the blood-brain barrier in such a short time may have caused a small transient activation of these components of the insulin signaling cascade, but the changes in neurotransmitter expression indicate a stimulation of the hypothalamus with the hormone. The diminished response of NPY to insulin in offspring from both groups of SE dams suggests maternal SE led to an impairment of hypothalamic insulin action. Loss of hypothalamic insulin signaling is sufficient to induce obesity and peripheral insulin resistance [66], [67]. Hypothalamic insulin action is an important contributor to the suppression of hepatic glucose production (24). The offspring of SE mothers showed significant glucose intolerance, which may be an early manifestation of impaired brain insulin signaling that could lead to a profound insulin resistance and type 2 diabetes later in life. Further long-term studies are required to determine the exact degree of deterioration of brain insulin signaling and its consequences on peripheral glucose homeostasis. However this may not be the only mechanism contributing to glucose intolerance in offspring from SE mothers. The phosphorylation of PKB in the liver is a crucial step leading to both suppression of hepatic glucose production and glucose uptake, and would be expected to occur after administration of exogenous insulin. Maternal exposure to smoke combined with HFD was sufficient to impair liver insulin action, as evidenced by a tendency to reduced phosphorylated/total PKB in insulin stimulated HFD-SE mice. Although the reduction did not reach statistical significance, it is likely that if animals are exposed to HFD after weaning such defects would be amplified and the liver would be the first tissue to show insulin resistance as seen in intrauterine growth retardation [68]. This suggests that changes in the liver may be contributing to the propensity to diabetes created by intrauterine SE [69].

The brain can sense the increase in blood glucose levels to inhibit liver glucose production. This regulation requires the glucose to be converted into lactate followed by stimulation of pyruvate metabolism [70]. Reduced capacity of brain glucose-lactate-pyruvate conversion and transport between astrocytes and neurons has been shown to diminish the negative feedback of gluconeogenesis leading to rising blood glucose level [70]. MCTs are involved in the transport of lactate and pyruvate between cells [71]. MCT4 and MCT2 are the predominant isoforms in astrocytes and neurons respectively [72]. Astrocytic MCT4 exports lactate produced during glycolysis into the extracellular space, from where MCT2 transports it into postsynaptic dendrites for mitochondrial oxidation [72]. In this study, MCT2 and MCT4 were reduced by both maternal HFD and SE, which may reduce the process of glucose-lactate-pyruvate metabolism, suggesting brain glucose metabolism is very sensitive to any adverse intrauterine nutritional change. This can further contribute to the glucose intolerance observed in these offspring.

In addition to nicotine, other products of cigarette smoke, such as carbon monoxide, can also directly affect the fetal brain [14]. Maternal nicotine exposure has been shown to reduce hypothalamic NPY and increase POMC expression in newborns [25]. The current study reveals that in the long term, hypothalamic NPY was only reduced in offspring from HFD-fed dams independent of maternal SE; however, Y1 receptor mRNA was significantly reduced by both maternal HFD consumption and SE. NPY activates several seven-transmembrane-domain G-protein-coupled receptors. The feeding stimulatory effect of NPY is primarily mediated through Y1 and Y5 receptors. Blockade of the Y1 receptor can eliminate the bulk of NPY-induced and fasting-induced feeding [73], [74], [75]. The reduced energy intake in chow-SE, HFD-sham and HFD-SE was mirrored by the downregulation in Y1 mRNA. Therefore, the Y1 receptor changes may directly contribute to reduced energy intake, even when NPY production was unchanged, such as in the SE-chow group. The Y5 receptor has been postulated to maintain the feeding response rather than initiate feeding in response to NPY [76]. On the other hand presynaptic Y2 receptors inhibit NPY release from the hypothalamus negatively regulating NPY [77]. Any additional contribution of these two receptors would require further investigation.

Adipose tissue derived leptin directly accesses the hypothalamus, reducing NPY and activating POMC to inhibit feeding and increase energy expenditure. In the current study, leptin was significantly increased in offspring from SE dams only, which was mainly due to increased production in white fat. Both smoking in humans and nicotine infusion in rodents increased plasma leptin concentration, as well as leptin mRNA expression in fat [78], [79]. Elevated plasma leptin remains after smoke cessation [78]. The increased plasma leptin and mRNA expression in chow-SE mice independent of fat mass could be directly due to intrauterine SE. This is offset by additional maternal HFD consumption in the HFD-SE group which requires further investigation. It seems that increased leptin level was only linked to increased hypothalamic expression of Sim1, lying downstream of POMC derived α-melanocyte-stimulating hormone, in the chow-SE group. However, here altered Sim1 was not link to any change in energy intake or body weight. The exact role of increased leptin secretion requires further investigation. Human studies suggest that maternal smoking can lead to increased immobility in offspring [29], [80], which could lead to significantly reduced energy expenditure. This may help to explain the discrepancy between reduced energy intake and adiposity observed in our study.

In summary, maternal cigarette SE and HFD consumption prior to, and during gestation and lactation led to faster growth during the suckling period and increased adiposity in adulthood in female offspring. Both exposures also caused disordered brain lactate transport. However, maternal SE played a stronger role in impairing glucose tolerance and brain insulin action. These findings offer a potential target for future interventions to reverse the detrimental maternal impact. Smoking cessation during pregnancy remains desirable to improve health outcomes in offspring.
